# A phase IIb randomized placebo-controlled trial testing the effect of MAG-EPA long-chain omega-3 fatty acid dietary supplement on prostate cancer proliferation

**DOI:** 10.1038/s43856-024-00456-4

**Published:** 2024-03-22

**Authors:** Karine Robitaille, Marie-Hélène Guertin, Afshin Jamshidi, Hui Wen Xu, Hélène Hovington, Jean-François Pelletier, Lisanne Beaudoin, Nikunj Gevariya, Louis Lacombe, Rabi Tiguert, Yves Caumartin, Thierry Dujardin, Paul Toren, Michele Lodde, Étienne Racine, Dominique Trudel, Martine Perigny, Thierry Duchesne, Josée Savard, Pierre Julien, Yves Fradet, Vincent Fradet

**Affiliations:** 1 CHU de Québec-Université Laval Research Center, Québec, QC G1R 3S1 Canada; 2 Centre de recherche sur le Cancer de l’Université Laval, Québec, QC G1R 3S3 Canada; 3 Institute of nutrition and functional foods (INAF) and NUTRISS Center - Nutrition, health and society of Université Laval, Québec, G1V 0A6 Canada; 4 Faculty of Medicine, Université Laval, Québec, QC, G1V 0A6 Canada; 5 Department of Mathematics and Statistics, Université Laval, Québec, QC G1V 0A6 Canada; 6 Centre de Recherche Clinique et Évaluative en Oncologie de L’Hôtel-Dieu de Québec, CHU de Québec-Université Laval, Québec, QC G1R 3S1 Canada; 7 Department of Pathology, CHU de Québec-Université Laval, Québec, QC G1R 2J6 Canada; 8 Centre de recherche du Centre Hospitalier de l’Université de Montréal (CRCHUM) et Institut du cancer de Montréal, and Department of Pathology and Cellular Biology, Université de Montréal, Montréal, H3C 3J7 Canada; 9 School of psychology, Université Laval, Montréal, QC G1R 2J6 Canada; 10 Present Address: Faculty of Medicine, Université Laval, Québec, QC, G1V 0A6 Canada

**Keywords:** Prostate cancer, Cancer therapy

## Abstract

**Background:**

High prostate eicosapentaenoic fatty acid (EPA) levels were associated with a significant reduction of upgrading to grade group (GG) ≥ 2 prostate cancer in men under active surveillance. We aimed to evaluate the effect of MAG-EPA long-chain omega-3 fatty acid dietary supplement on prostate cancer proliferation.

**Methods:**

A phase II double-blind randomized placebo-controlled trial was conducted in 130 men diagnosed with GG ≥ 2 prostate cancer and undergoing radical prostatectomy between 2015–2017 (Clinicaltrials.gov: NCT02333435). Participants were randomized to receive 3 g daily of either MAG-EPA (*n* = 65) or placebo (*n* = 65) for 7 weeks (range 4–10) prior to radical prostatectomy. The primary outcome was the cancer proliferation index quantified by automated image analysis of tumor nuclear Ki-67 expression using standardized prostatectomy tissue microarrays. Additional planned outcomes at surgery are reported including plasma levels of 27 inflammatory cytokines and fatty acid profiles in circulating red blood cells membranes and prostate tissue.

**Results:**

Cancer proliferation index measured by Ki-67 expression was not statistically different between the intervention (3.10%) and placebo (2.85%) groups (*p* = 0.64). In the *per protocol* analyses, the adjusted estimated effect of MAG-EPA was greater but remained non-significant. Secondary outcome was the changes in plasma levels of 27 cytokines, of which only IL-7 was higher in MAG-EPA group compared to placebo (*p* = 0.026). Men randomized to MAG-EPA prior to surgery had four-fold higher EPA levels in prostate tissue compared to those on placebo.

**Conclusions:**

This MAG-EPA intervention did not affect the primary outcome of prostate cancer proliferation according to nuclear Ki-67 expression. More studies are needed to decipher the effects of long-chain omega-3 fatty acid dietary supplementation in men with prostate cancer.

## Plain language summary

It is thought that our diet can impact our risk of cancer and affect outcomes in patients with cancer. Omega-3 fatty acids, mostly found in fatty fish, might be beneficial by protecting against prostate cancer and its adverse outcomes. We conducted a clinical trial to test the effects of an omega-3 dietary supplement (MAG-EPA) in men with prostate cancer. We randomly allocated 130 men to receive either MAG-EPA or a placebo for 7 weeks before their prostate cancer surgery. We measured a marker of how much tumor cells were proliferating (or growing in number) at the point of surgery, which might indicate how aggressive their disease was. However, the supplement did not affect tumor cell proliferation. The supplement was therefore not beneficial in this group of patients and further studies  are needed to test and confirm the effects of MAG-EPA on prostate cancer cells.

## Abstract (web summary)

Robitaille et al. report findings from a phase IIb randomized placebo-controlled trial evaluating the effect of a long-chain omega-3 fatty acid MAG-EPA dietary supplement on prostate cancer proliferation. The dietary supplement had no effect on the primary outcome of prostate cancer proliferation according to Ki-67 expression.

## Introduction

Globally every year, 1.4 M men are diagnosed with prostate cancer^1^, prompting important life-changing decisions as treatments harbor risks of adverse effects and are costly^2,3^. Lifestyle factors are thought to impact prostate cancer risk and progression^4^. A diet rich in long-chain omega-3 polyunsaturated fatty acids (LCω3), mainly found in seafood and fatty fish, could benefit prostate cancer patients, possibly by modulating tissue inflammation^5^. Observational studies have shown mixed results regarding associations between LCω3 and prostate cancer risk^6–8^. Epidemiologic data indicate that fish or marine-derived omega-3 fatty acids, which are traditional constituents of Asian diets, have a protective effect on prostate cancer survival^9^. Studies also suggest that marine-LCω3 may have more pronounced effects on biologically aggressive tumors or on their progression, and less pronounced effects on initiation or progression of more indolent prostate cancers^10–13^.

One of the most important determinants of prostate cancer aggressiveness and risk of progression is the cancer grade. Since 2014, the International Society of Urological Pathology (ISUP) agreed on a new system of five prostate cancer Grade-Groups (GG) based on the proportion of the three Gleason patterns 3, 4 or 5^14–16^. GG 1 tumors with only Gleason pattern 3 are indolent and most patients are recommended active surveillance. GG 2, 3 and 4 are defined by the proportion of pattern 4 (<50%, >50% and 100% respectively), and GG 5 have pattern 5. In a cohort of men with GG1 prostate cancer on active surveillance, we previously reported that prostate tissue levels of LCω3, particularly of eicosapentaenoic acid (EPA), were associated with a significant reduction of upgrading to GG ≥ 2 (detection of Gleason pattern 4) on the first follow-up biopsy^17,18^.

Humans cannot synthesize LCω3. Dietary intake is therefore required to increase blood levels, which can be challenged by the high levels of LCω6 in Western diet^19,20^. Supplements represent a quick and effective way to significantly increase blood levels of LCω3 which, upon insertion into cell membranes, increase their fluidity and trigger anti-inflammatory cascades^21,22^. EPA esterified in monoacylglycerides (MAG-EPA) is a molecule directly absorbed by enterocytes with no need to be hydrolyzed by pancreatic lipases unlike EPA esterified in ethyl ester^23,24^. Pharmacokinetic studies resulted in significantly higher EPA blood levels with MAG-EPA compared to those achieved using EPA esterified in ethyl ester^20,25^, a commonly studied supplement which was shown to reduce ischemic cardiovascular events in high risk patients^26^. In pre-clinical prostate cancer models, we showed that MAG-EPA significantly reduced prostate tumor growth compared to control supplements^27^.

The main hypothesis underlying the present trial was that a MAG-EPA supplement for 7 weeks before radical prostatectomy would increase EPA levels in prostatic tissue and potentially reduce the aggressiveness of prostate cancer. One suspected mechanism explaining this effect is through the reduction of the proliferative potential of cancer cells. Ki-67 is a nuclear protein only expressed during active phases of the cell cycle and not during quiescent state^28^. Ki-67 expression level by prostate cancer cells is used to quantify tumor proliferation, extrapolate tumor aggressiveness, and is increasingly proposed as a prognostic factor^29–31^. Moreover, a phase II randomized controlled trial comparing a low-fat diet enriched with fish oil to a western diet showed, in the fish-oil supplemented group, a significant reduction of prostate cancer Ki-67 expression, which was a secondary outcome of the trial^32^. Therefore, the primary outcome of this trial was to measure tumoral Ki-67 expression assessed at prostatectomy. The secondary endpoint reported herein was the effect of pre-surgical treatment on plasma levels of 27 inflammatory cytokines at prostatectomy. Additional planned secondary outcomes were the effect of a 12 month treatment of MAG-EPA versus placebo continued post-surgery on the patients’ psychological functioning and quality of life, which are reported elsewhere^33,34^. The pre-planned exploratory outcomes of fatty acid profiles in circulating red blood cells (RBC) membranes and prostate tissue at surgery are reported herein.

Overall, prostate cancer proliferation was not different between both groups. Only circulating interleukin (IL)-7 was higher in MAG-EPA group compared to placebo, while men randomized to MAG-EPA had four-fold higher EPA levels in prostate tissue compared to placebo.

## Methods

### Trial design

We conducted a double-blind phase IIb randomized placebo-controlled trial. Enrolled patients were randomized by the CHU de Québec-Université Laval clinical research oncology pharmacy to one of two arms with a 1:1 ratio using a computer-generated random listing with permuted random blocks of 2–8. The intervention consisted of six capsules of 625 mg of fish oil monoglycerides (each containing 500 mg of MAG-EPA) daily. This supplementation is a purified form of fish-oil that is highly concentrated in EPA giving a total dosage of three grams of EPA daily, as recommended by Health Canada for maintenance of general health^35^. The placebo was six capsules of high oleic sunflower oil (HOSO), giving 3.75 grams daily of HOSO. Placebo and MAG-EPA bottles and capsules were identical in appearance, odor and taste. Capsules were started 7 weeks in average (range 4–10) prior to radical prostatectomy and continued for 1-year post-prostatectomy. Patients were asked to maintain their usual diet.

At randomization, after verification of the randomized intervention by two pharmacists, patients received their capsules for a period of 3 months plus the delay to surgery. Capsules were prepared by the pharmacy according to a list of kit numbers generated by the manufacturer for placebo and MAG-EPA, and given to patients by the research nurse. Only the pharmacy staff was unblinded. Participants were instructed by the research nurse to take six capsules once a day (ideally at bedtime). Patients also received a reminder instruction sheet including the research nurse phone number in case of additional questions. Patients were not contacted between randomization and the first follow-up visit in clinic following surgery unless requested by them. No follow-up intervention was performed to reinforce overall compliance. Adherence was estimated by the pharmacist by pill count at the follow-up visit (after three months of surgery).

Secondary outcome of inflammatory mediators assessed in blood at prostatectomy is reported here. Other planned secondary outcomes such as psychological functioning and quality of life that were measured up to a year after surgery have been reported elsewhere in specialized journals^33,34^. Inflammatory mediators’ assessment in prostate tissue was not yet performed, due to cost and limited material.

Exploratory outcomes included fatty acids measurements in RBC membranes at study baseline and at surgery, as well as in prostate specimens collected at prostatectomy.

We also collected clinical data, as part of regular clinical follow-up of prostate cancer patients, such as PSA levels, cancer grade, cancer stage, biochemical recurrence, etc.

The study protocol was previously described in details^36^.

### Participants

Recruitment for this study occurred between February 12, 2015 and June 9, 2017 in the Centre Hospitalier Universitaire (CHU) de Québec—Université Laval. The trial was approved by our Institution’s Review Board (#2012–1012) and registered to *clinicaltrials.gov* (Effects of EPA on Prostate Cancer Cells Proliferation and Quality of Life (RCT-EPA)—NCT02333435). All patients provided written informed consent to participate in the study.

Participants were diagnosed with prostate cancer, Gleason score ≥ 7 (GG ≥ 2) for which radical prostatectomy was the chosen primary treatment. Patients were not eligible if they were allergic to fish/seafood or if they had bipolar disorder, since one secondary endpoint was psychological functioning. Patients already taking omega-3 supplements could participate after a wash out period of 8 weeks before randomization (*n* = 3). Other supplements had to be stopped for the whole intervention period.

### Baseline and follow-up information

At study baseline (randomization) and all along the trial, we collected fasting blood samples, anthropometry measurements (height, weight, waist circumference and skinfold thickness) and self-administered validated questionnaires about medical history and health behaviors, psychological/somatic symptoms and quality of life^37–39^. Information collected also include PSA, clinical data, education, smoking, exercise^40,41^, diet and alcohol consumption using a web-FFQ^42^ validated questionnaire^43^.

Adverse events that occurred in both study arms were collected systematically by the research nurse at the pre-prostatectomy visit and graded according to NCI Common Terminology Criteria for Adverse Events (CTCAE) (version 4.0)^44^.

### Outcomes

#### Assessment of Ki-67

We built tissue micro-arrays (TMA) of patient’s primary, secondary tumor grade regions and benign prostate tissue. One to three paraffin blocks containing tumor and/or normal tissue were selected for each patient. All tumor slides were examined and graded by a pathologist. Spots were selected from hematoxylin and eosin slides to represent the proportion of each Gleason pattern amongst dominant nodules and include a tertiary pattern when present in sufficient quantity. To build the TMA, four representative 1 mm tumor cores (two primary Gleason pattern cores and two secondary Gleason pattern cores) as well as two normal zones close to and away from the tumor (tumor-proximal and tumor-distal, respectively) were taken and placed on a recipient paraffin block along with appropriate alignment and controls using a tissue arrayer (Beecher Instruments, Sun Prairie, WI, USA).

Sections of five micron-thick were cut from the TMA blocks to perform immunohistochemistry (IHC) using a clinical pathology Dako platform. All sections were deparaffinized and heat-induced antigen retrieval with PT Link (PT-11#PT10027, Agilent) in Envision Flex Target Retrieval Solution High pH (#K8004, Agilent). Ready-to-use monoclonal mouse antibody clone MIB-1 was used for Ki-67 antigen (#IR626, Agilent) with Autostainer Link48 instrument (#AS480 Agilent) for 20 min. Slides were counter-stained with Harris hematoxylin. Digital images of IHC-stained TMA slides were obtained at 20X magnification using a slide scanner (NanoZoomer 2.0 HT; Hamamatsu, Bridgewater, NJ, USA).

The primary outcome for the study was nuclear expression of Ki-67 in tumor cells assessed as a percentage of tumor cells using an automated image analysis approach. We previously showed that this measure was associated with prostate cancer specific survival and highly correlated to an experienced duplicated manual counting^29^. Automated IHC analysis was performed using Calopix 3.2.0 software (TRIBVN healthcare, France). For the purpose of tissue recognition and segmentation, we used a morphometry algorithm by image learning to create a tissue mask and keeping only the tumor and normal epithelial component for analysis. Then, the Calopix «immuno-object» software was applied to each segment. The algorithm used allowed recognition of individual nuclei «objects» by isolating brown (DAB-stained) and blue (hematoxylin counter-stained) nuclei and reported their numbers. For each tissue core, the total number of objects detected (cell nuclei) and the percent of immunostained objects were computed. We performed five different algorithms to get a global Ki-67 score for each core. We then averaged the Ki-67 score of the patient’s TMA cores to get a total, primary, secondary, and benign tissue Ki-67 score for each patient. Ki-67 analysis were performed without knowledge of intervention group.

#### Inflammatory mediators

The plasma levels of 27 cytokines were measured using a 27-plex human cytokine kit (Bio-Plex Pro Human Cytokine 27-plex Assay from Bio-Rad #M500KCAF0Y), which measures levels of IL-1β, IL-1ra, IL-2, IL-4, IL-5, IL-6, IL-7, IL-8, IL-9, IL-10, IL-12 (p70), IL-13, IL-15, IL-17, eotaxin-1, bFGF, G-CSF, GM-CSF, IFN-γ, MCP-1 (MCAF), MIP-1α, MIP-1β, TNF-α, VEGF, IP-10, PDGF-BB, and RANTES, according to manufacturer’s instructions. Cytokine levels were measured at study baseline and at surgery. The Assays were run on Bio-Plex® 200 Systems and data analyzed using Bio-Plex Manager™ Software 6.1 Build 727. The changes of each cytokine between radical prostatectomy and study baseline was calculated, and the mean of change was reported for both groups.

#### Tissue levels of EPA in prostatectomy and RBC membranes

Fatty acid profiles in RBC were assessed at baseline and on the day of surgery. Fatty acid profiles were also assessed in snap frozen prostate tissue at surgery. Since a previous study in a similar population showed no difference between benign and malignant tissue fatty acid profiles^32^, prostate tissues for these analyses were all collected from the left anterior region of the prostate gland. Fatty acid profiles were determined by gas-chromatography mass-spectrometry after total lipid extraction according to a modified Folch method^45,46^ as described previously^47^. Briefly, lysates were isolated by centrifugation and washed with NaCl solution. A solution of chloroform/methanol 2:1 (v/v) was used to extract the lipids from both erythrocyte membranes and tumors with phosphatidylcholine C21:0 (Avanti Polar Lipids, Alabaster, AL), as an internal standard. Fatty acid profiles were obtained after methylation in methanol/benzene 4:1 (v/v) mixed with acetyl chloride^48^ and separation by capillary gas chromatography using a temperature gradient on a HP5890 gas chromatograph (Hewlett Packard, Toronto, Canada) equipped with a HP-88 capillary column (100 m x 0.25 mm i.d. x 0.20 mm film thickness; Agilent Technologies) coupled to a flame ionization detector. Identification of fatty acids were done according to retention time using standard mixtures: FAME 37 mix (Supelco Inc, Bellefonte, PA), GLC-411 FA mix (NuChek Prep Inc, Elysian, MN), as well as 22:5n-6 (Larodan AB, Malmö, Sweden) and 22:5n-3 (Supelco inc). Fatty acid content is expressed as relative percentages of total fatty acids for RBC and prostate tissue, as well as milligrams of fatty acids per gram of prostate tissue.

#### Clinical and pathological outcomes

As routinely performed, cancer grade and stage were assessed at study baseline (last biopsy leading to enrollment) and on prostatectomy specimens. Cancer grade was evaluated by a pathologist using the ISUP grading system^15,16^. Central pathology review of all biopsy and prostatectomy cases was performed by a dedicated uro-pathologist blinded on intervention group and on biopsy findings.

### Statistical analysis

The statistical analysis plan was previously published^36^. We used an intention-to-treat approach including all analyzable patients for whom a Ki-67 percentage could be obtained. Average Ki-67 expression from the four tumor cores at prostatectomy, expressed as a percentage of tumor cells, were compared between the intervention and placebo group using two-sided *T*-tests. Average Ki-67 expression from the two primary tumor cores and the two benign cores were also analyzed. Sensitivity analysis testing the individual Ki-67 analysis algorithms were conducted. Ki-67 measure was log-transformed because its distribution was positively skewed.

Multivariable linear regression analyses were performed to adjust for patients’ characteristics that were found to be unbalanced at study baseline (age, BMI, PSA, GG, clinical stage). Covariates were removed if they did not change the effect estimate of the study arm. All final models were adjusted for baseline GG, PSA and clinical T stage. Ki-67 was log-transformed and subtracting one to the exponentiation of the treatment effect corresponds to the Ki-67 relative change (%) in the treatment group compared to placebo. Multivariable linear regression models were fitted using the GLM procedure in SAS version 9.4 (SAS Institute, Cary, NC). *Per protocol* analyses were also performed among patients who adhered to the intervention and placebo by taking at least 80% of their capsules for the first three months for outcomes at prostatectomy.

Preplanned subgroup analyses by baseline LCω3 were not carried out because of missing values in the web-FFQ for some participants (23%). Subgroup analyses were therefore based on RBC fatty acids (%) at baseline which we showed to be correlated with diet in the previous month^18,43^. The median was used as a cut point. Analyses were also presented stratified by baseline biopsy grade. Subgroup analyses were performed using the same multivariable linear regression described above with the addition of an interaction term. Bilateral *p*-values of 0.05 were considered statistically significant.

For secondary outcome analysis, the change in cytokine levels were compared between groups using *T*-test, or the non-parametric Fligner-Policello test when data transformation to achieve normality was not possible. We had planned^36^ to use the Wilcoxon rank-sum test, but the variance and or the distribution shape was not similar for most cytokines. Thus, for simplicity, the Fligner-Policello test was used. We attempted to normalize the distribution of cytokine level data using simple approaches (e.g. Log(2), Log(10), square-root,…), box-plot^49,50^ and more complex procedures such as the COMBAT procedure using the “*sva*” and “*dbnorm*” R package^51,52^. The latter also accounts for batch effect correction. Unfortunately, none of these transformations reached the goal of normalizing the cytokine level data as tested using the Shapiro–Wilk test.

All data processing steps and statistical analyses were performed in the R v4.1.1 statistical environment (http://www.r-project.org).

### Sample size

Sample size analysis was done using a two-sample *t*-test for a log-normal geometric mean ratio with a two-sided significance level of 0.05, assuming equal variances, for the primary outcome, the percentage of tumor cells expressing Ki-67. In the study conducted by Aronson et al. ^32^, a statistically significant reduction of 32% in the proportion of cells expressing Ki-67 was observed in a group receiving a low-fat diet supplemented with fish oil compared to a control group assigned to a Western diet. We determined that, for the primary outcome, a total of 126 patients (63/group) will provide 90% power^53,54^ to detect a mean ratio of the proportions of cancer cells expressing Ki-67 of ≤0.8, i.e. a 20% difference across groups. Based on previous studies^32,55^, a coefficient of variation of 0.4 was assumed. Considering previous low trial dropout rates (<3%), the target sample size was established at 130 participants (65 per group).

### Reporting summary

Further information on research design is available in the Nature Portfolio Reporting Summary linked to this article.

## Results

**Fig. 1:**
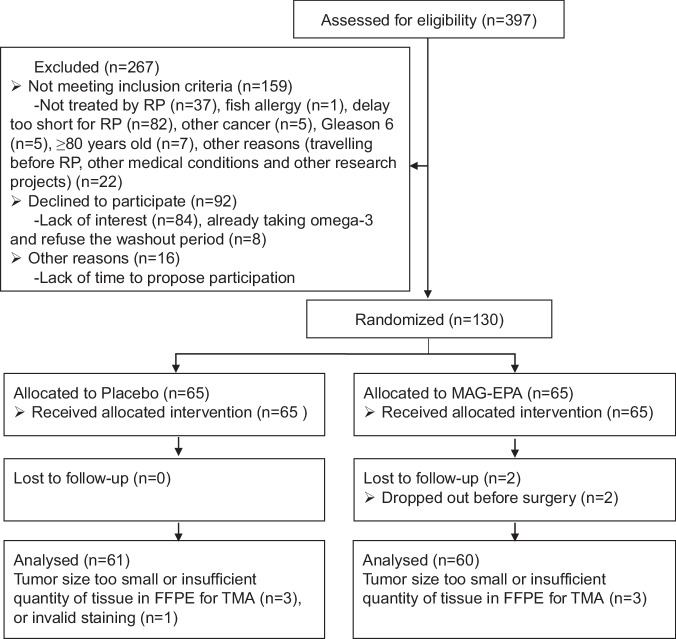
CONSORT trial flow diagram. RCT-EPA study flow chart for the primary endpoint (*n* = 121). For systemic inflammation (secondary endpoint), all randomized men have been included (*n* = 130). For adherence to intervention and adverse events, all men who underwent radical prostatectomy have been included (*n* = 128). MAG-EPA eicosapentaenoic acid monoacylglyceride, FFPE Formalin-fixed paraffin-embedded, TMA Tissue Microarray.

Participants were recruited between February 12, 2015 and June 9, 2017. Trial ended when the target sample size was reached. A total of 397 patients were assessed for eligibility (Fig. 1). Among them, 159 did not meet the inclusion criteria for reasons detailed in Fig. 1.

After randomization, only two patients dropped out (1.5%) before surgery in the intervention arm and three participants in each arm were also excluded because the tumor size was too small or an insufficient quantity of tissue was available in formalin-fixed paraffin-embedded (FFPE) prostatectomy for tissue microarray (TMA). One last participant of the placebo group was excluded from analysis because of invalid staining issue on TMA. The additional exclusions were not related to the intervention. A total of 61 patients in the placebo group and 60 patients in the intervention group were included in the *intention-to-treat* analysis for the primary endpoint.

All randomized patients were included for the reported secondary analysis. All patients who underwent radical prostatectomy were included in the exploratory analyses.

### Baseline characteristics

Baseline characteristics of participants are presented in Table 1. Compared to the group allocated to placebo, patients in the MAG-EPA intervention group were more likely to have a biopsy GG ≥ 4 (25.0% compared to 9.8%). Accordingly, prostate-specific antigen (PSA) values were, on average, higher in patients allocated to the intervention group (mean = 8.69 ng/mL) compared to patients allocated to placebo (mean = 6.69 ng/mL). Compared to placebo, patients in the intervention arm were older (64.9 vs 62.7 years’ old), had a slightly higher BMI (28.75 vs 27.45 kg/m^2^), and slightly lower EPA level in RBC membranes (0.73 vs 0.80 %). Other characteristics were similarly distributed across groups, including other fatty acid levels in RBC membranes at study baseline (Table 1).

**Table 1:** Characteristics of participants at study baseline

Characteristic	Placebo (*n* = 61)	MAG-EPA (*n* = 60)
**Cancer characteristics**		
PSA at randomization (ng/ml)		
Mean (Std)	6.69 (5.66)	8.69 (9.48)
Min, max	0.32, 39.00	0.83, 56.00
Median (Q1, Q3)	5.77 (4.05, 7.00)	6.11 (4.40, 8.81)
ISUP grade group, n (%)		
2 (Gleason 3 + 4)	37 (60.66)	28 (46.67)
3 (Gleason 4 + 3)	18 (29.51)	17 (28.33)
4 (Gleason 8)	5 (8.20)	11 (18.33)
5 (Gleason 9)	1 (1.64)	4 (6.67)
Cancer Stage *n* (%)		
T2a or less	56 (90.16)	48 (80.00)
T2b or T2c	3 (4.92)	4 (6.66)
T3 or more	3 (4.92)	8 (13.33)
NCCN risk, *n* (%)		
Intermediate	51 (83.61)	42 (70.00)
High	10 (16.39)	18 (30.00)
**Patient characteristics**		
Age (years)		
Mean (Std)	62.7 (7.44)	64.9 (6.39)
Body mass index (m/kg^2^)		
Mean (Std)	27.45 (4.43) *	28.74 (3.65) ^†^
Smoking status		
Current (%)	8 (13.11)	3 (5.00)
Past (%)	24 (39.34)	30 (50.00)
Never (%)	29 (47.54)	26 (43.33)
Missing value	0 (0.00)	1 (1.67)
Education, *n* (%)		
High school or less	18 (29.51)	23 (38.33)
Postsecondary diploma	24 (39.34)	16 (26.67)
University degree	18 (29.51)	19 (31.67)
Missing	1 (1.64)	2 (3.33)
**RBC fatty acid profile (%)** ^‡^		
Total ω3		
Mean (Std)	7.36 (0.97)	7.40 (1.15)
Median (Q1, Q3)	7.31 (6.70, 7.93)	7.25 (6.54, 8.08)
LCω3		
Mean (Std)	7.06 (0.98)	7.10 (0.23)
Median (Q1, Q3)	7.05 (6.47, 7.65)	6.90 (6.26, 7.74)
EPA		
Mean (Std)	0.80 (0.24)	0.73 (0.23)
Median (Q1, Q3)	0.79 (0.62, 0.90)	0.66 (0.57, 0.83)
Total ω6		
Mean (Std)	26.64 (1.30)	26.47 (1.50)
Median (Q1, Q3)	26.53 (25.69, 27.57)	26.71 (25.45, 27.41)
ω6:ω3 ratio		
Mean (Std)	3.70 (0.64)	3.68 (0.70)
Median (Q1, Q3)	3.61 (3.23, 4.08)	3.69 (3.21, 4.14)

^*^2 missing values. ^†^1 missing value. ^‡^RBC fatty acid profile is expressed as a percentage of total fatty acids. *Std* Standard deviation, *Q* Quartile, *NCCN* National Comprehensive Cancer Network, *PSA* Prostate-specific antigen, *RBC* Red blood cells, *ω3* Omega-3 fatty acids, *LCω3* Long chain omega-3 fatty acids, *MAG-EPA* monoacylglyceride-conjugated eicosapentaenoic acid, *ω6* Omega-6 fatty acids. *ISUP* International Society of Urological Pathology.

A validated web-based food frequency questionnaire (web-FFQ^42,43^) was completed by 100/130 (77%) of participants at study baseline. The mean intake of fat, carbohydrates, and proteins expressed as a percentage of total energy intake, was 33 ± 5%, 48 ± 7%, and 16 ± 2%, respectively, for an average daily energy intake of 2330 ± 745 Kcal. These values are in line with recent healthy dietary recommendations^56,57^. The mean omega-6:omega-3 dietary fatty acid ratio at baseline was 6.3 ± 1.8 which translated into a mean omega6:omega3 ratio in the RBC membranes of 3.7 ± 0.64.

### Intervention

The duration of the intervention prior to surgery was not different for both groups with an average duration of 52.0 ± 19.6 days for placebo group and 53.2 ± 37.4 days for MAG-EPA group (Table 2). Successful implementation of dosing regimen was similar between the two groups with 79% of patients in each group taking at least 80% of their capsules in the first three months of the study measured by pharmacy pill count. Adherence to intervention (% of taken pills) was similar across groups (87.5% placebo vs 90% MAG-EPA).

**Table 2:** Description of the intervention by study arm

Characteristics of the intervention	Placebo (*n* = 61)	MAG-EPA (*n* = 60)	*p*-value
Duration of intervention before RP (days) (mean ± Std)	52.0 ± 19.6	53.2 ± 37.4	0.82 ^§^
Successful implementation of dosing regimen* (%)	78.5 ^†^	79.4 ^‡^	0.83 ^||^
Adherence to intervention** (%) (mean ± Std)	87.5 ± 13.7	90.2 ± 14.8	0.30 ^§^
RBC EPA at time of RP (% of total fatty acid) (mean ± Std)	0.79 ± 0.39	2.77 ± 0.79	<0.0001 ^§^
Prostate tissue EPA at RP (% of total fatty acid) (mean ± Std)	0.26 ± 0.31	1.03 ± 0.46	<0.0001 ^§^

^*^Proportion of participants who underwent radical prostatectomy (RP) and took at least 80% of dose during the first 3 months. ^†^*n* = 65, including 2 patients with unknown adherence: remaining pills not returned to the pharmacy (*n* = 1), stopped intervention before 3 months (*n* = 1). ^‡^*n* = 63, including 7 patients with unknown adherence: remaining pills not returned to the pharmacy (*n* = 3), stopped intervention before 3 months (*n* = 1), never adhered to intervention (*n* = 1), no explanation (*n* = 2). **Proportion of dose taken by participants who underwent RP (*n* = 128), excluding patients with unknown adherence (*n* = 2 placebo; *n* = 7 MAG-EPA). ^§^*p*-value from independent samples *t*-test. ^||^*p*-value from Chi-square test. *RBC* red blood cells, *MAG-EPA* monoacylglyceride-conjugated eicosapentaenoic acid.

### Cancer proliferation index

#### Intention-to-treat analysis

Average Ki-67 expression in prostate tumor tissue was 3.10% in the intervention arm and 2.85% in the placebo arm (Table 3). The difference was not statistically significant (*p* = 0.66). Adjustment for GG, PSA and stage yielded a non-significant reduction of Ki-67 in the MAG-EPA arm (relative difference of −5.6%, 95%CI: −15.8 to 5.7, *p* = 0.64). Sensitivity analysis adjusting for age and BMI provided similar results. Analyses restricted to primary tumor cores for the Ki-67 assessment also provided similar non-significant results. Analyses stratified by baseline GG and levels of RBC omega-3 fatty acids (Supplementary Tables 1 and 2) showed that the intervention was similarly non-significant across all groups.

**Table 3:** Ki-67 expression, stratified by intervention group, *intention-to-treat* and *per protocol* analyses

	*Intention to treat*							
	Ki-67 expression (%)		Crude effect size			Adjusted effect size ^†^		
	Placebo	MAG-EPA	% difference in Ki-67	(95%CI)	*p*-value*	% difference in Ki-67	(95%CI)	*p*-value
**Total tumor Ki-67**	*n* = 61	*n* = 60	5.5	(-16.8, 33.8)	0.66	-5.7	(-24.7, 18.1)	0.64
mean (Std)	2.85 (1.74)	3.10 (2.12)						
**Primary tumor Ki-67**	*n* = 53	*n* = 48	6.2	(-18.6, 38.6)	0.66	-1.6	(-24.4, 28.0)	0.89
mean (Std)	2.99 (1.76)	3.15 (1.96)						
**Benign prostate tissue**	*n* = 62	*n* = 59	-0.4	(-26.2, 34.4)	0.98	-2.5	(-28.5, 32.9)	0.70
mean (Std)	0.58 (0.55)	0.59 (0.54)						
	***Per protocol*** ^‡^							
	**Ki-67 expression (%)**		**Crude effect size**			**Adjusted effect size** ^†^		
	**Placebo**	**MAG-EPA**	**% difference in Ki-67**	**(95%CI)**	*p*-value*	**% difference in Ki-67**	**(95%CI)**	***p-value***
**Total tumor**	*n* = 47	*n* = 46	-1.1	(-25.3, 31.0)	0.94	-14.0	(-34.0, 12.1)	0.41
mean (Std)	2.90 (1.77)	3.00 (2.08)						
**Primary tumor**	*n* = 42	*n* = 37	-0.3	(-27.0, 36.1)	0.98	-11.5	(-34.7, 20.0)	0.60
mean (Std)	3.00 (1.74)	3.11 (2.15)						
**Benign prostate tissue**	*n* = 48	*n* = 45	-5.0	(-32.7, 34.1)	0.77	-9.2	(36.6, 30.0)	0.45
mean (Std)	0.54 (2.55)	0.53 (0.48)						

Effect size: Ki-67 was log-transformed and the percent difference of the Ki-67 expression (%) in the MAG-EPA group compared to placebo is estimated by the following: (exp(βinterv)-1) x 100. *p-value from independent samples t-test. ^†^ Multivariable linear regression models adjusted for ISUP grade group (2, 3 and 4 + ), PSA, and clinical stage at the biopsy before surgery. The adjusted *p*-value is calculated using generalized linear model (GLM) for the effect of the intervention. ^‡^Included participants with a compliance of ≥80% of capsules consumed over the first 3-month period. MAG-EPA: monoacylglyceride-conjugated eicosapentaenoic acid.

#### Per protocol analysis

Among participants taking at least 80% of study capsules, the average Ki-67 expression in prostate tumor tissue was 3.00% in the intervention arm and 2.90% in the placebo arm (*p* = 0.94, Table 3).

Adjusted analyses showed a non-significant Ki-67 reduction in the MAG-EPA arm (relative difference of −14.0%, 95%CI: −34.0 to 12.1, *p* = 0.41). Pre-planned analyses stratified by baseline GG and levels of RBC omega-3 fatty acids (Supplementary Tables 3 and 4) showed that the intervention was similarly non-significant across all groups.

### Systemic inflammatory profile

**Fig. 2:**
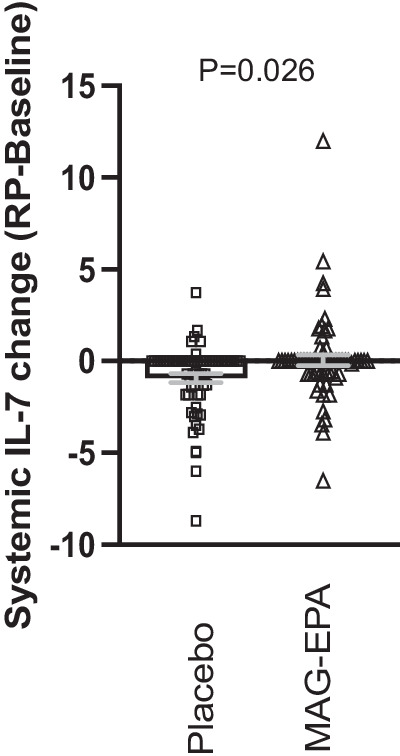
Effects of MAG-EPA on interleukin-7 (IL-7) cytokine level in enrolled men. Difference of IL-7 level (pg/mL) after 7 weeks of intervention and study baseline. Baseline: *n* = 130; RP: *n* = 126 (2 patients in placebo group did not have blood collection at RP, 2 patients in MAG-EPA dropped out before surgery). Gray bars are the mean ± SEM. *P*-value from Fligner-Policello test between placebo and MAG-EPA groups. RP radical prostatectomy, MAG monoacylglyceride, EPA eicosapentaenoic acid.

As planned secondary outcome, inflammatory-related markers were profiled in the plasma of participants at study baseline and at surgery (Table 4). Baseline levels were similar between both groups for all cytokines measured (Supplementary Table 5). From the 27 cytokines measured, only the change of IL7 level after 7 weeks of intervention was significantly different between MAG-EPA and placebo groups (Fig. 2, *p* = 0.026). However, this difference was not significant among participants taking at least 80% of study capsules (*per protocol* analysis, *p* = 0.055, Supplementary Table 5).

**Table 4:** Systemic inflammatory profile after 7 weeks of intervention, stratified by intervention group, intention-to-treat analysis

	Mean difference (std) between RP and baseline (pg/mL)		
Cytokines	Placebo (*n* = 63) *	MAG-EPA (*n* = 63) *	*p*-value
IL-1b	-0.27 (0.38)	-0.30 (0.45)	0.66 ^†^
IL-1ra	-18.69 (46.46)	-13.41 (65.11)	0.50 ^†^
IL-2	-1.07 (1.35)	-0.85 (1.35)	0.32 ^†^
IL-4	-0.40 (0.55)	-0.26 (0.62)	0.20 **
IL-5	-1.04 (2.07)	-0.89 (4.53)	0.13 ^†^
IL-6	-0.27 (0.80)	1.69 (13.37)	0.14 ^†^
IL-7	-0.85 (1.87)	0.04 (2.35)	0.026 ^†^
IL-8	-0.64 (1.99)	1.01 (10.84)	0.15 ^†^
IL-9	2.35 (23.88)	-1.88 (64.40)	0.73 ^†^
IL-10	-0.38 (0.71)	-0.42 (1.12)	0.88 ^†^
IL-12p70	-0.39 (0.77)	-0.75 (3.37)	0.87 ^†^
IL-13	-0.92 (1.23)	-1.02 (1.27)	0.62 ^†^
IL-15	-0.11 (2.32)	4.33 (30.85)	0.41 ^†^
IL-17	-0.55 (1.62)	-0.56 (2.15)	0.46 ^†^
Eotaxin	-4.58 (9.56)	-2.98 (10.51)	0.37 **
bFGF	-5.30 (9.69)	-5.95 (10.11)	0.57 ^†^
G-CSF	-3.07 (13.72)	1.98 (15.02)	0.06 ^†^
GM-CSF	-0.19 (0.62)	0.13 (3.57)	0.55 ^†^
INFg	-0.98 (1.27)	-2.85 (15.09)	0.63 ^†^
MCP-1	-2.15 (4.74)	-0.31 (4.47)	0.06 ^†^
MIP-1a	-0.05 (0.29)	-0.07 (0.54)	0.94 ^†^
MIP-1b	7.41 (22.41)	6.88 (21.34)	0.56 ^†^
TNFa	-3.23 (6.50)	-2.75 (5.51)	0.57 ^†^
VEGF	-12.20 (33.93)	-19.66 (48.69)	0.98 ^†^
IP-10	-69.42 (226.30)	-111.28 (246.90)	0.79 ^†^
PDGF	82.26 (249.85)	107.19 (312.54)	0.61 ^†^
Rantes	852.65 (2356.60)	974.85 (2298.04)	0.65 ^†^

^*^The sample size is greater than for the main Ki-67 analysis as all patients are included. *N* = 126: 2 patients in MAG-EPA group dropped out before surgery and 2 patients in placebo group did not have blood collection at prostatectomy. *P*-value from **independent samples *t*-test or ^†^ FIigner Policello test. *MAG-EPA* monoacylglyceride-conjugated eicosapentaenoic acid, *MD* mean difference, *RP* radical prostatectomy.

### Effect of the intervention on RBC and tissue EPA content

**Fig. 3:**
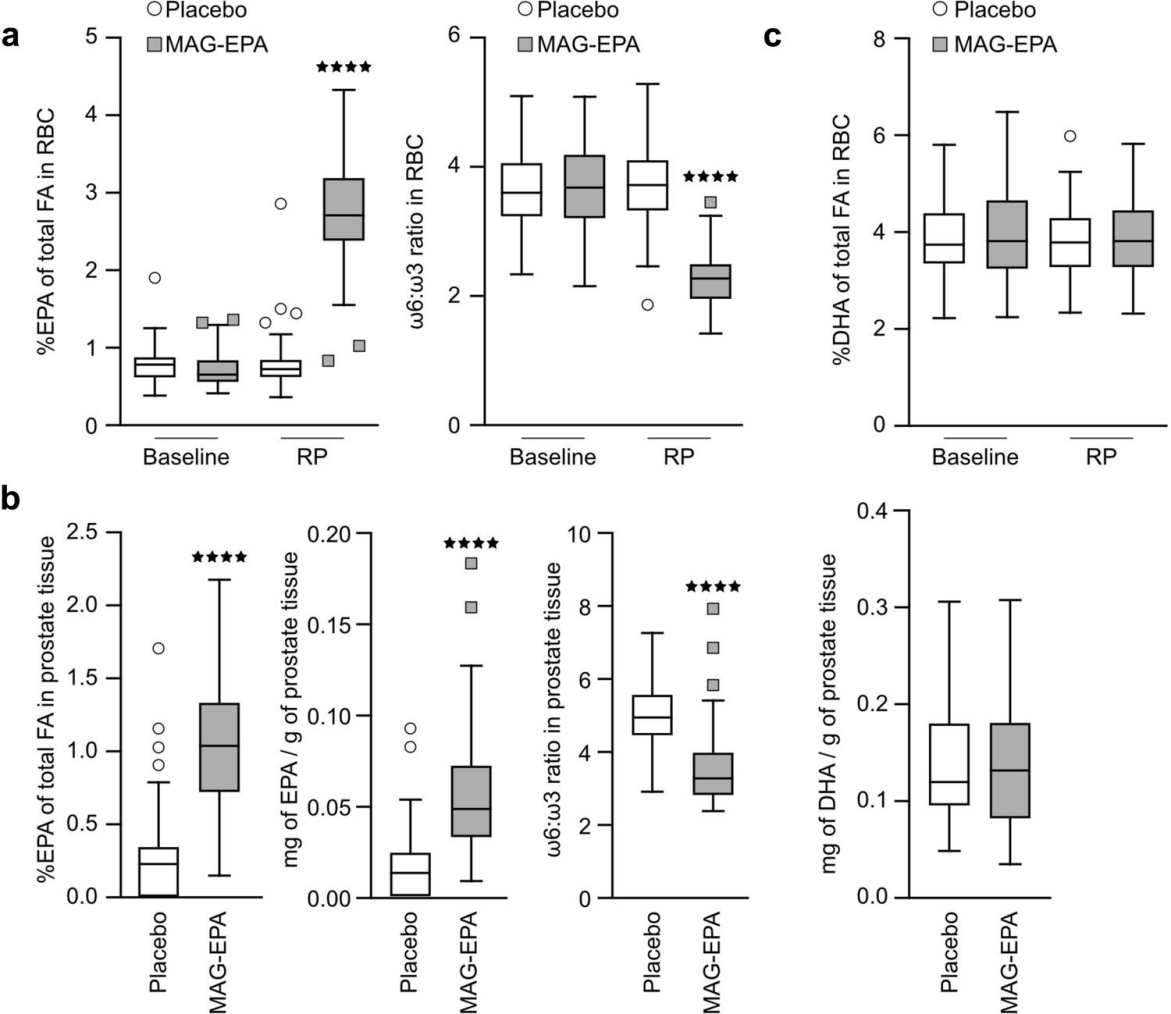
Effects of MAG-EPA on fatty acid profiles in enrolled men. **a** Fatty acid levels of red blood cell (RBC) membranes (% of total fatty acid content). Baseline: *n* = 121; RP: *n* = 118 (2 patients did not have blood collection at RP, 1 patient withdrew consent for additional research). Left: relative level of EPA, and Right: ω6:ω3 ratio. **b** Fatty acid levels in prostate tissue, *n* = 117 (3 patients did not have tissue collected for research, 1 patient withdrew consent for additional research). Left: relative EPA level (% of fatty acid content), Middle: absolute EPA level (mg of fatty acids per gram of tissue), and Right: ω6:ω3 ratio. **c** DHA level of RBC membranes (% of total fatty acid content, left) and of prostate tissue at RP (mg of DHA per g of tissue, right). Error bars are ± SEM. ****p < 0.0001, student *T*-test between placebo and MAG-EPA group, unpaired, two-tailed. ω3 omega-3 fatty acids, ω6 omge-6 fatty acids, RP radical prostatectomy, MAG-EPA eicosapentaenoic acid monoacylglyceride, Q quartile. FA fatty acids.

At prostatectomy, the EPA content (% of total fatty acids) in RBC membranes was significantly higher in the intervention group (2.77% in MAG-EPA group versus 0.79% in placebo, *p* < 0.0001) as well as in the prostate tissue (1.03% in MAG-EPA versus 0.26% in placebo, *p* < 0.0001) (Table 2, Fig. 3a, b), while the docosahexaenoic acid (DHA) content was unchanged (Fig. 3c, *p* = 0.81).

### Pathological outcomes

Clinical outcomes at prostatectomy are presented in Table 5. In contrast to baseline biopsy, prostatectomy grading was more similar between groups.

**Table 5:** Clinical outcomes at prostatectomy

	Placebo	MAG-EPA
	*N* = 65 *	*N* = 63 *
PSA mean (Std)	6.10 (5.34)	7.90 (8.76)
ISUP Grade Group, n (%)		
1 (Gleason 6)	1 (1.5)	3 (4.8)
2 (Gleason 3 + 4)	35 (53.9)	27 (42.9)
3 (Gleason 4 + 3)	23 (35.4)	23 (36.5)
4 (Gleason 8)	2 (3.1)	5 (7.9)
5 (Gleason 9)	4 (6.2)	5 (7.9)
Pathological stage, n (%)		
T2 or less (N0)	44 (67.7)	42 (66.7)
T3 or T4 (N0)	15 (23.1)	13 (20.6)
N+ (any T)	6 (9.2)	8 (12.7)

^*^The sample size is greater than for the main Ki-67 analysis as all patients who underwent radical prostatectomy are included.

*p*-value from ^†^independent samples *t*-test, ^‡^ordinal logistic regression comparing the change over the intervention time between groups, adjusted for PSA, ISUP grade group (2, 3 and 4+), and clinical stage at the biopsy before surgery. *PSA* prostate-specific antigen, *ISUP* International Society of Urological Pathology, *MAG-EPA* monoacylglyceride-conjugated eicosapentaenoic acid.

### Adverse events

MAG-EPA intervention for an average of 7 weeks before surgery was well tolerated and resulted in low rate of reported side effects (Table 6). There was no significant difference in the number of patients reporting side effects between the placebo (5/65 or 7.7%) and the MAG-EPA (7/63 or 11.1%) arms of the trial. However, 1 patient on the placebo versus 5 patients (including two before prostatectomy) on the MAG-EPA stopped taking the supplement because of adverse events during the first three months of the study.

**Table 6:** Reported adverse effects

	Placebo (*n* = 65) * n (%)	MAG-EPA (*n* = 63) * *n* (%)
Diarrhea	1 (1.5)	2 (3.2)
Skin rash	2 (3.1)	1 (1.6)
Nausea	1 (1.5)	3 (4.8)
Digestive problems	1 (1.5)	1 (1.6)
Total patients with events	5 (7.7) **	7 (11.1) **

^*^The sample size is greater than for the main Ki-67 analysis as all patients who underwent radical prostatectomy are included. **1 patient in placebo group and 5 patients in MAG-EPA group stopped study supplementation because of adverse effects.

*MAG-EPA* monoacylglyceride-conjugated eicosapentaenoic acid.

## Discussion

In this trial, EPA supplementation did not reduce prostate cancer cell proliferation measured by the Ki-67 index at prostatectomy compared to placebo. A greater effect magnitude was observed in the *per protocol* analyses, but the differences remained not significant. Compared to placebo, the change in IL-7 level was higher in MAG-EPA group. We showed that a daily dose of three grams of MAG-EPA during 7 weeks on average significantly increased fourfold the EPA level in prostate tissue compared to placebo.

This study showed that MAG-EPA supplement intervention is feasible, tolerated and acceptable to patients diagnosed with localized prostate cancer before radical prostatectomy. Most participants who were treated by prostatectomy (*n* = 128) had a successful implementation of dosing regimen (79% taking at least 80% of their capsules in the first 3 months of study) and good adherence to intervention (% of taken pills) despite the absence of follow-up contacts during the intervention. This low-intensity compliance follow-up in our trial is closer to real world practice and contrasts with a previous pre-prostatectomy trial of shorter duration (31 days versus 52 days in our trial) whereby patients “were contacted weekly by study staff to reinforce compliance”^32^. EPA levels in RBC membranes and prostate tissue, both increased following the intervention, which also support the feasibility of MAG-EPA supplementation. Changing individuals’ diet is difficult, hence the popularity of dietary supplements in the general population. To our knowledge, this is the first study measuring the modulation of a specific fatty acid subtype at the absolute level, not only relative level, in the targeted prostate tissue.

The effect of an omega-3 diet on prostate cancer proliferation was previously studied by Aronson et al. ^32^ in a phase II randomized controlled trial comparing a low-fat diet enriched with fish oil to a controlled western diet for 4–6 weeks, in 55 prostate cancer patients undergoing radical prostatectomy^32^. As a secondary outcome, Ki-67 expression was significantly lower in the low-fat fish oil-supplemented group compared to the western diet group. An important difference between that trial and ours is related to the diet. In the Aronson trial, the control group was under a supervised western diet trying to achieve a 15:1 omega6:omega3 fatty acid ratio, suspected to be deleterious for prostate cancer, while the control was a neutral placebo in our trial. The mean omega-6:omega-3 dietary fatty acid ratio at baseline was 6.3 ± 1.8 in our cohort compared to 9.6 pre-intervention in their trial^32^. This omega-6:omega-3 ratio in RBC stayed the same for the placebo group overtime (Fig. 2a), suggesting that the participants did not change their diet during the course of our trial. The healthier diet in our study population may have decreased the power to detect a significant effect of MAG-EPA supplementation on tumor Ki-67 expression. The omega-6:omega-3 ratio decreased to 2.2 after the MAG-EPA intervention, similar to the fish oil based intervention in the Aronson trial (ratio of 2.7). In the latter study, Ki-67 was expressed in 3.9% of prostate tumor cells for the fish oil intervention group compared to 6.2% in the western diet group. In our study, Ki-67 was expressed in 2.9% and 3.1% of prostate tumor cells in placebo and MAG-EPA groups, respectively. This suggests that increased Ki-67 expression could be more related to unhealthy LCω6 rich diet, rather than to a LCω3 intervention and that omega-6 fatty acids could drive Ki-67 increased expression.

Based on fatty acid profiling in prostate tissue, the absolute level of EPA fatty acid subtype was increased following the concentrated MAG-EPA intervention compared to placebo, but not DHA level. This confirms that the conversion of EPA into DHA in humans is very slow, especially in men tissues, as suggested previously^58^. According to previous results using fish oil-based intervention^32,59^, DHA fatty acid subtype could also be a more important modulator than EPA for the Ki-67 specific marker.

Biological effects of LCω3 are thought to be mediated, at least in part, by their anti-inflammatory properties. We profiled 27 circulating inflammatory mediators, and compared the changes of cytokine levels following 7 weeks of intervention. Only IL-7 was higher in MAG-EPA group compared to placebo, suggesting that MAG-EPA could enhance IL7-driven immunity. IL-7 is known to play a role in both B and T cells’ differentiation, development, and maturation during adaptive immunity. It was also shown to have antitumor activity by enhancing the proliferation and survival of T lymphocytes in the tumor microenvironment^60^. Indeed recent studies support a potential effect of systemic administration of IL-7 to improve immunotherapy response in prostate cancer^61,62^. However, we cannot exclude that this only difference was due to multiple comparisons. The significant difference was also weaker in the *per protocol* analysis, suggesting at best a modest effect of MAG-EPA on circulating IL-7.

How high EPA prostate tissue level may affect prostate cancer aggressiveness remains unclear. We previously showed that MAG-EPA treatment reduced cancer growth in the TRAMP-C2 syngeneic immune-competent mouse model associated with a significant downregulation of angiogenesis- and vascular-related pathways in the tumor, as well as significant decreased of tumor blood vessel diameter^27^. Prostate tumor blood vessel diameter was also significantly reduced in the first subset of ten MAG-EPA treated, compared to the first 10 placebo treated patients from the present study, but not in the adjacent normal tissue, suggesting that the anti-angiogenic effect is on the tumor-specific microenvironment^27^. Using the same TRAMP-C2 model, we observed a reduced tumor growth in mice fed with an LCω3 enriched diet compared to controls which was associated with an increase in tumor tissue levels of EPA and its derived metabolites, a reduction of pro-angiogenic prostaglandin E2 and increased levels of F4-neuroprostanes and resolvins suspected to have anti-inflammatory effects^63^. More recent data from a subset of patients of the present trial suggest a beneficial effect of MAG-EPA supplement on the gut microbiome^64^.

Some limitations are worth mentioning. We did not stratify the randomization for cancer grade. The MAG-EPA group had more cases of higher-grade prostate cancer which increased the Ki-67 expression. Adjusted analyses controlled for this difference but residual confounding remains possible. Our study population had a favorable omega-6:omega-3 dietary ratio to start with, as discussed above. It is possible that the MAG-EPA intervention could have reduced the Ki-67 index if performed in a population of prostate cancer patients with higher omega-6 and lower omega-3 intake, which presumably would have had a higher baseline prostate cancer proliferative rate.

In conclusion, pre-operative MAG-EPA dietary supplementation did not reduce cancer cell Ki-67 index. More studies are needed to better understand the biological and clinical outcomes following this concentrated EPA supplementation and determine if and how it can benefit prostate cancer patients.

### Supplementary information

Supplementary Information

Description of Additional Supplementary Files

Supplementary Data

Reporting summary

## Data Availability

All source data underlying the graphs and charts are available as Supplementary Data accessible in the Supplementary Information section. The datasets generated with individual deidentified participant data, including data dictionaries, and codes used during the current study are available from the corresponding author on reasonable request. Data will be available following the publication with no end date for researchers who submit a proposal to the corresponding author. Data can be used to achieve aims in the approved proposal and will be available after signature of a data access agreement. Study protocol is available in open access from the *BMC Cancer* website (https://bmccancer.biomedcentral.com/articles/10.1186/s12885-017-3979-9).
