# Supplementary material for: A phase IIb randomized placebo-controlled trial testing the effect of MAG-EPA long-chain omega-3 fatty acid dietary supplement on prostate cancer proliferation
Source: Commun Med (Lond). 2024 Mar 22;4:56. doi: 10.1038/s43856-024-00456-4 (PMC10960033; doi:10.1038/s43856-024-00456-4)
Supplement: Supplementary file 1 — Supplementary Information [file 43856_2024_456_MOESM1_ESM.pdf]

Supplementary Table 1 Ki-67 expression stratified by baseline grade group (post-hoc stratification), intention-to-treat analysis.

	Ki-67 expression (%)		
**Grade Group at Baseline**	**Placebo**	**MAG-EPA**	**p-values***
**Total tumor**			
**ISUP Grade Group 2**<br>mean (Std)	**n = 37**<br>2.39 (1.41)	**n = 28**<br>2.43 (1.57)	0.98
**ISUP Grade Group 3**<br>mean (Std)	**n = 18**<br>3.17 (1.76)	**n = 17**<br>2.70 (1.64)	0.35
**ISUP Grade Group 4 or 5**<br>mean (Std)	**n = 6**<br>4.71 (2.34)	**n = 15**<br>4.77 (2.65)	0.94
**Primary tumor**			
**ISUP Grade Group 2**<br>mean (Std)	**n = 30**<br>2.69 (1.55)	**n = 20**<br>2.49 (1.36)	0.74
**ISUP Grade Group 3**<br>mean (Std)	**n = 17**<br>3.26 (1.96)	**n = 16**<br>2.71 (1.44)	0.61
**ISUP Grade Group 4 or 5**<br>mean (Std)	**n = 6**<br>3.66 (2.19)	**n = 12**<br>4.84 (2.50)	0.31
**Benign prostate tissue**			
**ISUP Grade Group 2**<br>mean (Std)	**n = 38**<br>0.62 (0.63)	**n = 27**<br>0.45 (0.40)	0.09
**ISUP Grade Group 3**<br>mean (Std)	**n = 18**<br>0.53 (0.43)	**n = 17**<br>0.66 (0.55)	0.29
**ISUP Grade Group 4 or 5**<br>mean (Std)	**n = 6**<br>0.39 (0.27)	**n = 15**<br>0.74 (0.68)	0.25

*p-values from t-test for the log-transformed Ki-67 proliferation index.
MAG-EPA: monoacylglyceride-conjugated eicosapentaenoic acid.

Supplementary Table 2 Ki-67 expression stratified by prespecified omega-3 subgroups, intention-to-treat analysis.

		Crude analyses		
**Tissue**	**Baseline characteristics**	**Ki-67 expression (%) Mean (Std)**		**p-value †**
		**Placebo**	**MAG-EPA**	
**Total tumor**				
	**Total RBC ω3 ***	n = 61	n = 60	
	Low (5.53-7.27)	2.53 (1.77)	2.42 (1.44)	0.90
	Adequate (>7.27-10.55)	3.16 (1.68)	3.87 (2.50)	0.45
	**Total RBC LCω3 ***	n = 61	n = 60	
	Low (5.22-6.97)	2.60 (1.76)	2.42 (1.44)	0.91
	Adequate (>6.97-10.28)	3.08 (1.71)	3.87 (2.50)	0.31
**Primary tumor**				
	**Total RBC ω3 ***	n = 53	n = 48	
	Low (5.53-7.27)	2.75 (1.74)	2.60 (1.42)	0.99
	Adequate (>7.27-10.55)	3.21 (1.79)	3.85 (2.35)	0.40
	**Total RBC LCω3 ***	n = 53	n = 48	
	Low (5.22-6.97)	2.83 (1.72)	2.60 (1.42)	0.80
	Adequate (>6.97-10.28)	3.12 (1.81)	3.85 (2.35)	0.29

* Fatty acids are expressed as a percentage of total fatty acids. Stratification is based on median fatty acid value (below or equal to median; above median). † p-value from t-test.
MAG-EPA: monoacylglyceride-conjugated eicosapentaenoic acid; RBC: red blood cells; ω3: omega-3 fatty acids; LCω3: long-chain omega-3 fatty acids (EPA+DPA+DHA).

Supplementary Table 3 Ki-67 expression stratified by baseline grade group (post-hoc stratification), per protocol analysis.

	**Ki-67 expression (%)**		
**Grade Group at Baseline**	**Placebo**	**MAG-EPA**	**p-values***
**Total tumor**			
**ISUP Grade Group 2**<br>mean (Std)	**n = 31**<br>2.46 (1.42)	**n = 23**<br>2.49 (1.72)	0.76
**ISUP Grade Group 3**<br>mean (Std)	**n = 12**<br>3.36 (2.08)	**n = 11**<br>2.18 (1.15)	0.14
**ISUP Grade Group 4 or 5**<br>mean (Std)	**n = 4**<br>4.91 (2.02)	**n = 12**<br>4.73 (2.46)	0.79
**Primary tumor**			
**ISUP Grade Group 2**<br>mean (Std)	**n = 27**<br>2.67 (1.53)	**n = 17**<br>2.51 (1.48)	0.70
**ISUP Grade Group 3**<br>mean (Std)	**n = 11**<br>3.63 (2.13)	**n = 10**<br>2.11 (1.38)	0.16
**ISUP Grade Group 4 or 5**<br>mean (Std)	**n = 4**<br>3.54 (1.78)	**n = 10**<br>5.14 (2.51)	0.28
**Benign prostate tissue**			
**ISUP Grade Group 2**<br>mean (Std)	**n = 32**<br>0.55 (0.60)	**n = 22**<br>0.37 (0.27)	0.08
**ISUP Grade Group 3**<br>mean (Std)	**n = 12**<br>0.58 (0.49)	**n = 11**<br>0.52 (0.30)	0.65
**ISUP Grade Group 4 or 5**<br>mean (Std)	**n = 4**<br>0.31 (0.11)	**n = 12**<br>0.80 (0.75)	0.15

*p-values from t-test for the log-transformed Ki-67 proliferation index.
MAG-EPA: monoacylglyceride-conjugated eicosapentaenoic acid.

Supplementary Table 4 Ki-67 expression stratified by prespecified omega-3 subgroups, per protocol analysis.

		**Crude analyses**		
**Tissue**	**Baseline characteristics**	**Ki-67 expression (%) Mean (Std)**		**p-value †**
		**Placebo**	**MAG-EPA**	
**Total tumor**				
	**Total RBC ω3 ***	n = 47	n = 46	
	Low (5.53-7.27)	2.50 (1.73)	2.21 (1.18)	0.81
	Adequate (>7.27-10.55)	3.22 (1.78)	3.93 (2.52)	0.57
	**Total RBC LCω3 ***	n = 47	n = 46	
	Low (5.22-6.97)	2.50 (1.73)	2.21 (1.18)	0.81
	Adequate (>6.97-10.28)	3.22 (1.78)	3.93 (2.52)	0.57
**Primary tumor**				
	**Total RBC ω3 ***	n = 42	n = 37	
	Low (5.53-7.27)	2.72 (1.64)	2.48 (1.45)	0.68
	Adequate (>7.27-10.55)	3.25 (1.83)	3.93 (2.65)	0.58
	**Total RBC LCω3 ***	n = 42	n = 37	
	Low (5.22-6.97)	2.72 (1.64)	2.48 (1.45)	0.68
	Adequate (>6.97-10.28)	3.25 (1.83)	3.93 (2.65)	0.58

* Fatty acids are expressed as a percentage of total fatty acids. Stratification is based on median fatty acid value (below or equal to median; above median). † p-value from t-test.
MAG-EPA: monoacylglyceride-conjugated eicosapentaenoic acid; RBC: red blood cells; ω3: omega-3 fatty acids; LCω3: long-chain omega-3 fatty acids (EPA+DPA+DHA).

Supplementary Table 5 Circulating cytokine levels of participants à study baseline.

Cytokine	Mean (std) level (pg/mL) Placebo (n = 65)	MAG-EPA (n = 65)	p-value *
IL-1b	0.62 (0.67)	0.61 (0.50)	0.34
IL-1ra	39.32 (46.75)	46.81 (74.70)	0.78
IL-2	1.89 (1.48)	2.22 (2.47)	0.59
IL-4	1.88 (0.82)	1.91 (1.00)	0.93
IL-5	2.18 (3.29)	5.90 (23.15)	0.60
IL-6	1.03 (0.79)	1.36 (2.20)	0.92
IL-7	2.29 (3.48)	2.22 (3.69)	0.76
IL-8	3.43 (1.97)	4.09 (7.42)	0.97
IL-9	58.26 (31.43)	69.02 (95.13)	0.82
IL-10	2.26 (1.40)	2.20 (1.54)	0.83
IL-12p70	0.79 (0.84)	2.73 (12.43)	0.24
IL-13	1.41 (1.77)	1.41 (1.52)	0.82
IL-15	4.69 (13.69)	15.38 (55.01)	0.51
IL-17	4.77 (1.62)	5.25 (2.61)	0.58
Eotaxin	39.66 (18.55)	38.33 (17.79)	0.88
bFGF	18.06 (7.58)	19.79 (13.12)	0.53
G-CSF	28.74 (34.72)	31.26 (39.87)	0.95
GM-CSF	0.42 (0.79)	0.93 (2.74)	0.61
INFg	1.26 (1.15)	9.92 (64.71)	0.58
MCP-1	15.48 (19.22)	12.93 (7.83)	0.75
MIP-1a	1.29 (1.58)	1.49 (2.15)	0.41
MIP-1b	58.56 (26.65)	61.52 (29.21)	0.82
TNFa	16.33 (8.73)	16.49 (7.04)	0.63
VEGF	20.24 (41.66)	37.36 (66.68)	0.28
IP-10	380.08 (227.46)	463.42 (479.29)	0.87
PDGF	224.27 (155.29)	278.96 (294.01)	0.48
Rantes	2927.50 (1745.38)	2677.10 (1548.68)	0.35

*P-value from Fligner Policello test.
MAG-EPA: monoacylglyceride-conjugated eicosapentaenoic acid.

Supplementary Table 6 Systemic inflammatory profile after seven weeks of intervention, stratified by intervention group, per protocol analysis.

Cytokine	Mean difference (std) between RP and baseline (pg/mL)		p-value
	Placebo (n = 50) *	MAG-EPA (n = 49) *	
IL-1b	-0.27 (0.38)	-0.33 (0.48)	0.40 †
IL-1ra	-20.04 (45.77)	-15.69 (58.41)	0.24 †
IL-2	-1.05 (1.32)	-0.88 (1.40)	0.43 †
IL-4	-0.41 (0.59)	-0.24 (0.57)	0.15 **
IL-5	-0.92 (2.13)	-1.16 (5.09)	0.65 †
IL-6	-0.28 (0.89)	2.02 (15.16)	0.56 †
IL-7	-0.86 (1.90)	-0.06 (2.43)	**0.055** †
IL-8	-0.67 (2.14)	-0.3 (2.45)	0.20 †
IL-9	2.16 (25.49)	-3.08 (72.38)	0.66 †
IL-10	-0.39 (0.72)	-0.38 (0.78)	0.95 **
IL-12p70	-0.34 (0.73)	-0.77 (3.79)	0.67 †
IL-13	-0.86 (1.23)	-1.07 (1.15)	0.22 †
IL-15	-0.11 (1.55)	3.58 (32.31)	0.46 †
IL-17	-0.58 (1.51)	-0.70 (2.09)	0.48 †
Eotaxin	-4.66 (10.23)	-2.33 (10.12)	0.26 **
bFGF	-4.95 (9.85)	-6.95 (9.95)	0.25 †
G-CSF	-2.25 (14.08)	0.39 (11.95)	0.25 †
GM-CSF	-0.15 (0.53)	0.22 (4.01)	0.60 †
INFg	-0.91 (1.31)	-3.62 (17.05)	0.57 †
MCP-1	-2.11 (5.00)	-0.43 (4.49)	0.08 **
MIP-1a	-0.05 (0.31)	-0.02 (0.34)	0.69 †
MIP-1b	7.63 (22.75)	8.28 (20.35)	0.40 †
TNFa	-3.14 (7.11)	-2.46 (5.40)	0.45 †
VEGF	-10.10 (34.79)	-25.00 (44.50)	0.20 †
IP-10	-76.02 (250.97)	-65.65 (114.36)	0.37 †
PDGF	76.39 (246.81)	93.46 (318.19)	0.76 †
Rantes	995.25 (2467.75)	1081.00 (2245.24)	0.85 **

* Included participants with a compliance of >=80% of capsules consumed over the first 3 months period. P-value from ** independent samples T-test or † Fligner Policello test. MAG-EPA: monoacylglyceride-conjugated eicosapentaenoic acid; MD: mean difference; RP: radical prostatectomy.
