# Supplementary material for: A phase IIb randomized placebo-controlled trial testing the effect of MAG-EPA long-chain omega-3 fatty acid dietary supplement on prostate cancer proliferation
Source: Commun Med (Lond). 2024 Mar 22;4:56. doi: 10.1038/s43856-024-00456-4 (PMC10960033; doi:10.1038/s43856-024-00456-4)
Supplement: Supplementary file 2 — Description of Additional Supplementary Files [file 43856_2024_456_MOESM2_ESM.pdf]

Description of Additional Supplementary Files

File name: Supplementary Data 1
Description: This file contains all source data underlying the graphs and charts.
