# Supplementary material for: A phase IIb randomized placebo-controlled trial testing the effect of MAG-EPA long-chain omega-3 fatty acid dietary supplement on prostate cancer proliferation
Source: Commun Med (Lond). 2024 Mar 22;4:56. doi: 10.1038/s43856-024-00456-4 (PMC10960033; doi:10.1038/s43856-024-00456-4)
Supplement: Supplementary file 4 — Reporting summary [file 43856_2024_456_MOESM4_ESM.pdf]

nature portfolio

Corresponding author(s): Fradet, Vincent

Last updated by author(s): Jan 17, 2024

Reporting Summary

Nature Portfolio wishes to improve the reproducibility of the work that we publish. This form provides structure for consistency and transparency in reporting. For further information on Nature Portfolio policies, see our Editorial Policies and the Editorial Policy Checklist.

Statistics

For all statistical analyses, confirm that the following items are present in the figure legend, table legend, main text, or Methods section.

n/a	Confirmed	
☐	☒	The exact sample size (*n*) for each experimental group/condition, given as a discrete number and unit of measurement
☐	☒	A statement on whether measurements were taken from distinct samples or whether the same sample was measured repeatedly
☐	☒	The statistical test(s) used AND whether they are one- or two-sided<br>*Only common tests should be described solely by name; describe more complex techniques in the Methods section.*
☐	☒	A description of all covariates tested
☐	☒	A description of any assumptions or corrections, such as tests of normality and adjustment for multiple comparisons
☐	☒	A full description of the statistical parameters including central tendency (e.g. means) or other basic estimates (e.g. regression coefficient) AND variation (e.g. standard deviation) or associated estimates of uncertainty (e.g. confidence intervals)
☐	☒	For null hypothesis testing, the test statistic (e.g. *F*, *t*, *r*) with confidence intervals, effect sizes, degrees of freedom and *P* value noted<br>*Give P values as exact values whenever suitable.*
☒	☐	For Bayesian analysis, information on the choice of priors and Markov chain Monte Carlo settings
☒	☐	For hierarchical and complex designs, identification of the appropriate level for tests and full reporting of outcomes
☐	☒	Estimates of effect sizes (e.g. Cohen's *d*, Pearson's *r*), indicating how they were calculated

Our web collection on statistics for biologists contains articles on many of the points above.

Software and code

Policy information about availability of computer code

Data collection	We used standard data collection, storage and protection policies. These are well described in the M&M section.
Data analysis	We used standard commercially available software and data modeling strategies. These are well described in the M&M section. Code can be made available if judged useful.

For manuscripts utilizing custom algorithms or software that are central to the research but not yet described in published literature, software must be made available to editors and reviewers. We strongly encourage code deposition in a community repository (e.g. GitHub). See the Nature Portfolio guidelines for submitting code & software for further information.

Data

Policy information about availability of data

All manuscripts must include a data availability statement. This statement should provide the following information, where applicable:

- Accession codes, unique identifiers, or web links for publicly available datasets
- A description of any restrictions on data availability
- For clinical datasets or third party data, please ensure that the statement adheres to our policy

We have included a data availability statement: "All source data underlying the graphs and charts are available as Supplementary Data accessible in the Supplementary Information section. The datasets generated with individual deidentified participant data, including data dictionaries, and codes used during the current study are available from the corresponding author on reasonable request. Data will be available following the publication with no end date for researchers

who submit a proposal to the corresponding author. Data can be used to achieve aims in the approved proposal and will be available after signature of a data access agreement. Study protocol is available in open access from the BMC Cancer website (https://bmccancer.biomedcentral.com/articles/10.1186/s12885-017-3979-9)."

Human research participants

Policy information about studies involving human research participants and Sex and Gender in Research.

Reporting on sex and gender	The biological sex is a consideration for this project since only men develop prostate cancer and are therefore eligible for the first lane of recruitment in this study. Since gender refers to socially constructed identities, it was not specifically considered in this project.
Population characteristics	Participants were men diagnosed with prostate cancer, Gleason score ≥7 (ISUP grade group ≥2) for which radical prostatectomy was the chosen primary treatment.
Recruitment	The trial protocol was offered to all consecutive patients treated at our center once radical prostatectomy treatment was chosen. We observed expected participation rate, without any outlying clinical characteristics of study participants versus patients treated outside of thistrial.
Ethics oversight	Research Ethics Board of CHU de Québec - Université Laval

Note that full information on the approval of the study protocol must also be provided in the manuscript.

Field-specific reporting

Please select the one below that is the best fit for your research. If you are not sure, read the appropriate sections before making your selection.

☒ Life sciences ☐ Behavioural & social sciences ☐ Ecological, evolutionary & environmental sciences

For a reference copy of the document with all sections, see nature.com/documents/nr-reporting-summary-flat.pdf

Life sciences study design

All studies must disclose on these points even when the disclosure is negative.

Sample size	The target sample size was established based on the set primary endpoint of the percentage of tumor cells expressing Ki67. The sample size calculations determined that a total of 126 patients (63 per group) was necessary to detect a 20% reduction in the percentage of tumor cells expressing Ki67. Based on previous studies, a coefficient of variation of 0.4 was assumed. Considering previous low trial dropout rates (<3%), the target sample size was established at 130 participants (65 pergroup).
Data exclusions	We included all data points that was possible to include. For the primary outcome, we excluded 9 patients in whom it was not possible to obtain tumor tissue for staining (n=8), and whom staining failed (n=1). For the secondary endpoint, all patients were included.
Replication	To increase precision of the primary outcome measurement, we performed five different algorithms for the automatic nuclear Ki67 quantification. The main outcome result was the average of the five algorithms. In addition, two independent statisticians performed the same analyses. The results were concordant.
Randomization	Group allocation was performed only after inclusion criteria were verified centrally. Randomization sequence used a 1:1 allocation scheme and was computer generated by the clinical research oncology pharmacy using permuted random block size of 2 to 8.
Blinding	Allocation group was kept concealed, only the pharmacy staff was unblinded to the intervention. Investigators, patients, nurses and analysts were all blinded. The blinding was also kept during data analysis.

Reporting for specific materials, systems and methods

We require information from authors about some types of materials, experimental systems and methods used in many studies. Here, indicate whether each material, system or method listed is relevant to your study. If you are not sure if a list item applies to your research, read the appropriate section before selecting a response.

Materials & experimental systems

n/a	Involved in the study
☐	☒ Antibodies
☒	☐ Eukaryotic cell lines
☒	☐ Palaeontology and archaeology
☒	☐ Animals and other organisms
☐	☒ Clinical data
☒	☐ Dual use research of concern

Methods

n/a	Involved in the study
☒	☐ ChIP-seq
☒	☐ Flow cytometry
☒	☐ MRI-based neuroimaging

Antibodies

Antibodies used	FLEX Monoclonal Mouse Anti-Human Ki-67 Antigen, Clone MIB-1, Ready-to-Use (Link), Unconjugated, (#IR626, Agilent)
Validation	The antibody used in this manuscript is commercially available with standardized validation and routinely used by our clinical pathology lab. The image analyze algorithms were developed, validated and published by our team (Desmeules et al. Diagnostic Pathology, 2015. DOI 10.1186/s13000-015-0294-0)

Clinical data

Policy information about clinical studies

All manuscripts should comply with the ICMJE guidelines for publication of clinical research and a completed CONSORT checklist must be included with all submissions.

Clinical trial registration	NCT02333435
Study protocol	Research Ethics Board approved the protocol written in French, as required by our institution. The study protocol was also approved in English lately. The study protocol was published (Guertin et al. BMC Cancer 2018. DOI10.1186/s12885-017-3979-9).
Data collection	Well described in M&M section. All patients were treated at CHU de Québec - Univeristé Laval between Feb 2015 to June2017.
Outcomes	All reported outcomes reported herein are pre-specified at prostatectomy. The primary outcome was nuclear Ki67 tumor expression. The secondary outcome reported here was the systemic inflammation biomarkers at surgery. Additional secondary outcomes (quality of life and psychosocial functioning) are beyond the scope of this manuscript and are reported elsewhere. Inflammatory mediators' assessment in prostate tissue was not yet performed, due to cost and limited material.
